# Editorial: Foot-and-Mouth Disease in Swine

**DOI:** 10.3389/fvets.2017.00133

**Published:** 2017-08-21

**Authors:** Andres M. Perez, Preben W. Willeberg

**Affiliations:** ^1^Department of Veterinary Population Medicine, College of Veterinary Medicine, University of Minnesota, Saint Paul, MN, United States; ^2^National Veterinary Institute, Technical University of Denmark, Lyngby, Denmark

**Keywords:** foot-and-mouth disease, swine, epidemiology, pathogeny

**Editorial on the Research Topic**

**Foot-and-Mouth Disease in Swine**

## Introduction

Foot-and-mouth disease (FMD) is one of the most devastating diseases of livestock (1). The disease is caused by infection with a picornavirus, generically referred as FMD virus (FMDV), which is considered one of the most infectious agents affecting animals (2). FMD status affects national and international movement and trade of animals and animal products, and food animal trade is expected to play an important role in poverty alleviation (Perez). Applied knowledge about FMD pathogenesis and epidemiology is important in the design and implementation of effective prevention and control programs, minimizing detrimental effects of FMD outbreaks. Decision tools have been developed by applying simulation models based on characteristics of FMD pathogenesis and epidemiology. These tools are meant to be used by risk managers and risk communicators to help prioritize control options during an FMD epidemic and making the evidence available for all stakeholders [Willeberg et al.; (3)].

Much of the literature on FMD has focused on the pathogenesis and epidemiology of the disease in cattle. However, FMD also affects other food animal species, most notably, swine. This research topic contributes to the gain and dissemination of important knowledge on the dynamics of one of the most devastating diseases of livestock when occurring in the pig, a susceptible species for which limited information is available in the peer-reviewed literature. The ultimate objective of these original articles and reviews was to contribute preventing and mitigating the impact of FMD in swine, thus, promoting health and economic development of non-affected as well as affected countries and regions.

This research topic features nine studies supplementing the state-of-the-art of the knowledge on the pathogenesis and epidemiology of FMD in swine. Three papers focus on the analysis of experimental studies, which have been designed with the objective of gaining basic knowledge on the pathogenesis of the disease. Three other papers summarize the results of field studies and review fundamental features of FMD transmission and the effectiveness of FMD vaccination in swine. The last three papers describe the design and implementation of applied epidemiology approaches to prevent or mitigate the impact of FMD epidemics in disease-free regions.

## Experimental Studies

The potential for FMDV transmission during the preclinical incubation period of infection was assessed in seven groups of pigs, which were sequentially exposed to a group of infected donor pigs (Stenfeldt et al.). Results demonstrated significant differences between contact-exposed groups, in the time between virus exposure to first detection of FMDV shedding, viremia, and clinical lesions. These results are important because they suggest that FMDV shedding in oropharyngeal fluids does not correlate well with clinical signs of FMDV infection in pigs, which may affect FMDV transmission, and hence the effectiveness of control strategies in the face of an FMD epidemic.

The extent to which maternally derived antibodies interfere with the protection conferred by FMD vaccination was assessed in piglets (Dekker et al.). Results suggest that immune responses in piglets with maternally derived antibodies vaccinated at 7 or 9 weeks of age are similar to those of piglets without maternal immunity that were vaccinated at 3 weeks of age. These results are important because they demonstrate that maternally derived antibody levels in piglets strongly depend on the antibody titer in the sow, so the optimal time for vaccination in piglets will be affected by the vaccination scheme and the quality of vaccine used in the sows.

A review of results from recent experimental studies suggested that pigs were more susceptible to FMDV infection *via* exposure of the upper gastrointestinal tract (oropharynx) than through virus inhalation (Stenfeldt et al.). Due to massive amplification and shedding of virus, acutely infected pigs constitute an important reservoir for amplification of virus over the course of an epidemic. However, infection is ultimately cleared due to a strong humoral response and there is no evidence of subclinical persistence of FMDV infection in pigs. In general, FMDV infection in pigs spreads rapidly among in-contact pigs and efficiency of transmission depends on a number of factors, including the virus strain and the intensity of exposure to the virus. Under experimental conditions, physical separation of pigs may be sufficient to prevent virus transmission, which, in the field, may result in different infection patterns between and among sections or rooms within pig farms.

## Descriptive Studies and Reviews

Foot-and-mouth disease is still to be eradicated from many regions of the world; for example, FMD epidemics are recurrent in Israel and in many Middle Eastern countries (Elnekave et al.). Although, for cultural reasons, swine production is not prevalent in the Middle East, there is a large population of wild boars in the region. On assessing 120 wild boar (*Sus scrofa lybicus*) samples, 15 (12.5%) were found to be FMD seropositive. Most of the FMD-positive samples obtained from wild boar [13/15 (86.7%)] were collected during 2007, and because clinical signs of FMD infection were not evident in these animals, it is possible that, under certain conditions, wild boars may contribute to maintenance and spread of FMD infection in the region.

Foot-and-mouth disease control programs in endemic settings are largely based on the use of vaccines. However, recent FMD epidemics in Asia demonstrated that developing an adequate artificial immune response is challenging in pigs. The performance of FMDV vaccines has been reviewed to identify knowledge gaps and provide ideas to improve efficiency and efficacy of vaccination programs (Lyons et al.). Factors found to affect vaccine performance include potency, antigenic payload, formulation of the vaccine, antigenic match between the vaccine and heterologous circulating field strains, and the vaccine administration regime, i.e., timing, frequency, and herd-level coverage.

In countries free from FMD infection, such as the US, response strategies are required in early control of hypothetical incursions, and disease simulation models play a role in the design of prevention and mitigation activities. Values associated with the duration of the stages of FMD infection (latent period, subclinical period, incubation period, and duration of infection), the probability of transmission (within-herd and between-herd *via* spatial spread), and the diagnosis of FMD within a herd were evaluated using a combination of a meta-analysis of the peer-reviewed literature and elicitation of expert opinion (Kinsley et al.). Although most US swine practitioners believed that they could detect an FMD incursion relatively soon, some estimated that up to half of the herd would need to show clinical signs before detection *via* passive surveillance would occur, which suggests the need for disease awareness programs in FMD-free countries.

## Applied Studies

The ultimate objective of epidemiological studies is to create the foundations for the design and implementation of strategies and policy to prevent or mitigate disease impact, including modeling and risk analysis techniques [Perez; Willeberg et al.; (3)]. The risk of introducing FMDV into Australia through illegal importation of infected meat was quantified for large-scale pig producers, small-scale producers (<100 sows) selling at sales yards and abattoirs, and small-scale producers selling through informal means (Hernández-Jover et al.). Risk was quantified using scenario trees and Monte Carlo stochastic simulation. Although risk was predicted to be extremely low for the three sectors of the pig industry, exposure through direct swill feeding was 10–100 times more likely to occur than through contact with infected feral pigs. Furthermore, the FMDV would be more likely to spread from small-scale farms selling at sales yards and abattoirs compared to other sectors. Factors most influential on the probability of FMDV spread from the first-case farm included the effectiveness of the farmer in early disease detection, the probability of FMDV spread through contaminated fomites, and contact with ruminants on the farm. These results stress again the importance of programs to facilitate awareness and promote early detection of the disease in the face of an epidemic, and also, the importance of biosecurity in preventing disease introduction and spread into FMD-free areas.

One of the most challenging aspects of FMD response plans in FMD-free countries is the design of plans to secure continuity of business (COB) while implementing control measures to keep the food system functional and mitigate the impact of the epidemic. Animal health emergency response plans have been designed in the US to mitigate the unintended negative consequence of an FMD epidemic to stakeholders (Goldsmith et al.; Patterson et al.). The COB principles and goals adopted by the United States Department of Agriculture for responding to foreign animal diseases, such as FMD, are to (1) detect, control, and contain the disease in animals as quickly as possible; (2) to eradicate the disease using strategies that stabilize animal agriculture, the food supply, and the economy that protect public health and the environment; and (3) to provide science- and risk-based approaches and systems to facilitate COB for non-infected animals and non-contaminated animal products. A protocol has been developed to use proactive risk assessments (i.e., before an outbreak happens) to authorize specific movements from low-risk premises located in control areas that are not known to be infected (Goldsmith et al.). However, this requires a system of prioritization of different types of movements. Highest priority was given by the industry to movement of weaned pigs originating from multiple sow farm sources to an off-site nursery or wean-to-finish facility, the movement of employees or commercial crews, the movement of vaccination crews, the movement of dedicated livestock hauling trucks, and the movement of commercial crews such as manure haulers and feed trucks onto, off, or between sites. These critical movements provide an initial guide for prioritization of risk management efforts and resources to be better prepared in the event of an FMD outbreak in the US and other FMD-free countries with the ultimate objective of regaining disease-free status while mitigating the impact on the industry.

## Final Remarks

In summary, the articles in this research topic explore and discuss important aspects of FMDV infection in swine, highlighting features that differ from traditional knowledge on the pathogenesis and epidemiology of the disease, as observed in cattle. The research topic advances our understanding of challenges in the design and implementation of vaccination campaigns to control the disease, the importance of biosecurity measures to prevent and limit its spread, and the role that modeling and risk assessments may play in mitigating the economic impact of FMD epidemics in swine.

## Author Contributions

AP and PW co-edited the research topic and wrote this editorial.

## Conflict of Interest Statement

The authors declare that the research was conducted in the absence of any commercial or financial relationships that could be construed as a potential conflict of interest.
